# Supplementary material for: Time-Series Comparative Transcriptome Analyses of Two Potato Cultivars with Different Verticillium Wilt Resistance
Source: Plants (Basel). 2025 Dec 21;15(1):26. doi: 10.3390/plants15010026 (PMC12787827; doi:10.3390/plants15010026)
Supplement: Supplementary file 1 [file plants-15-00026-s001.zip › plants-3981279suppl/supplV2_20251027.pdf]

Supplementary Information for

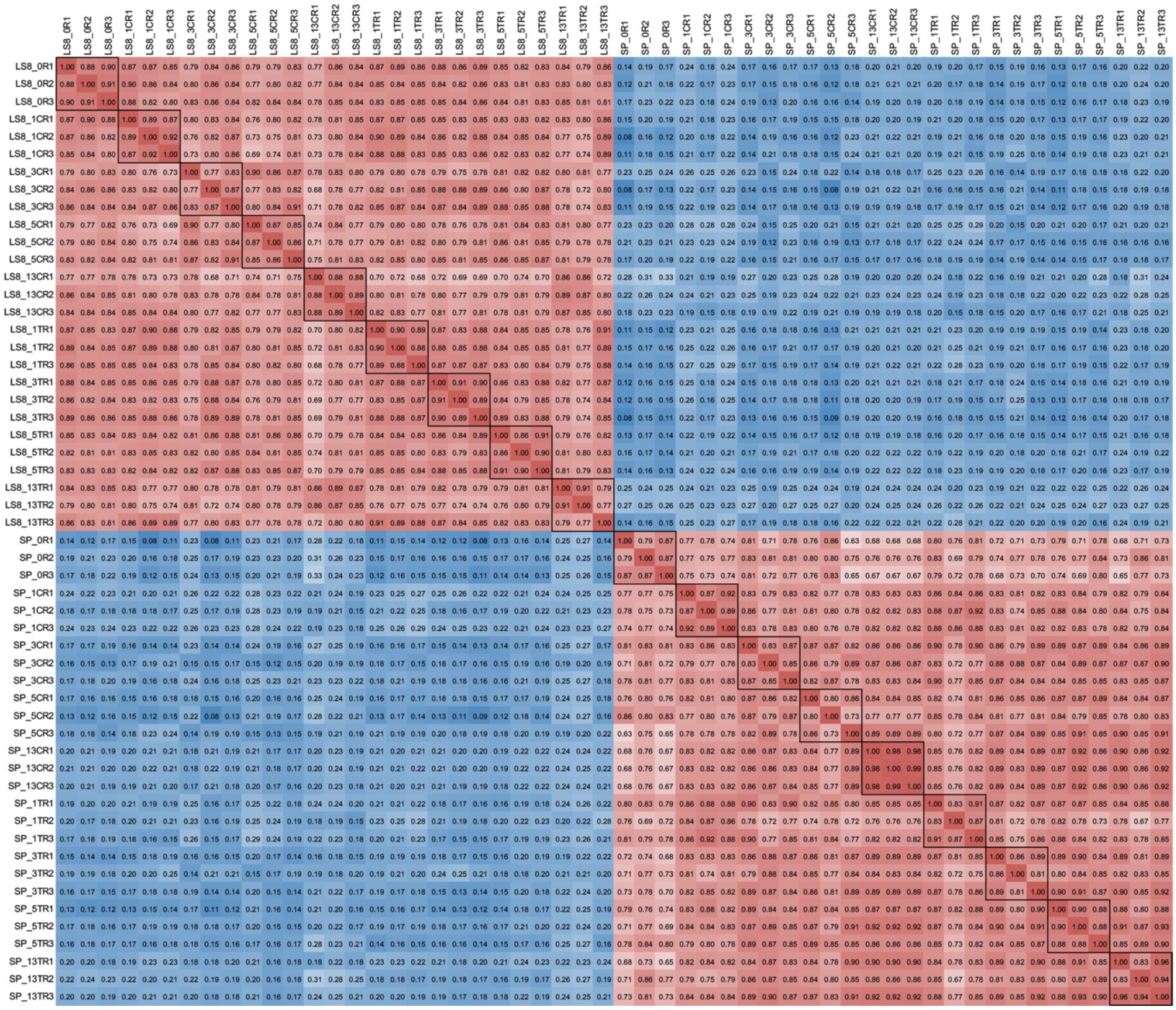

Supplementary Figure S1. The analysis of correlation between the replicated RNA-seq samples. The Pearson correlation coefficient (PCC) was calculated between RNA-seq samples, with those between the replicates highlighted with black boxes. The PCC values are shown in heat map, with blue and red colors representing the low and high PCC values (from 0 to 1).

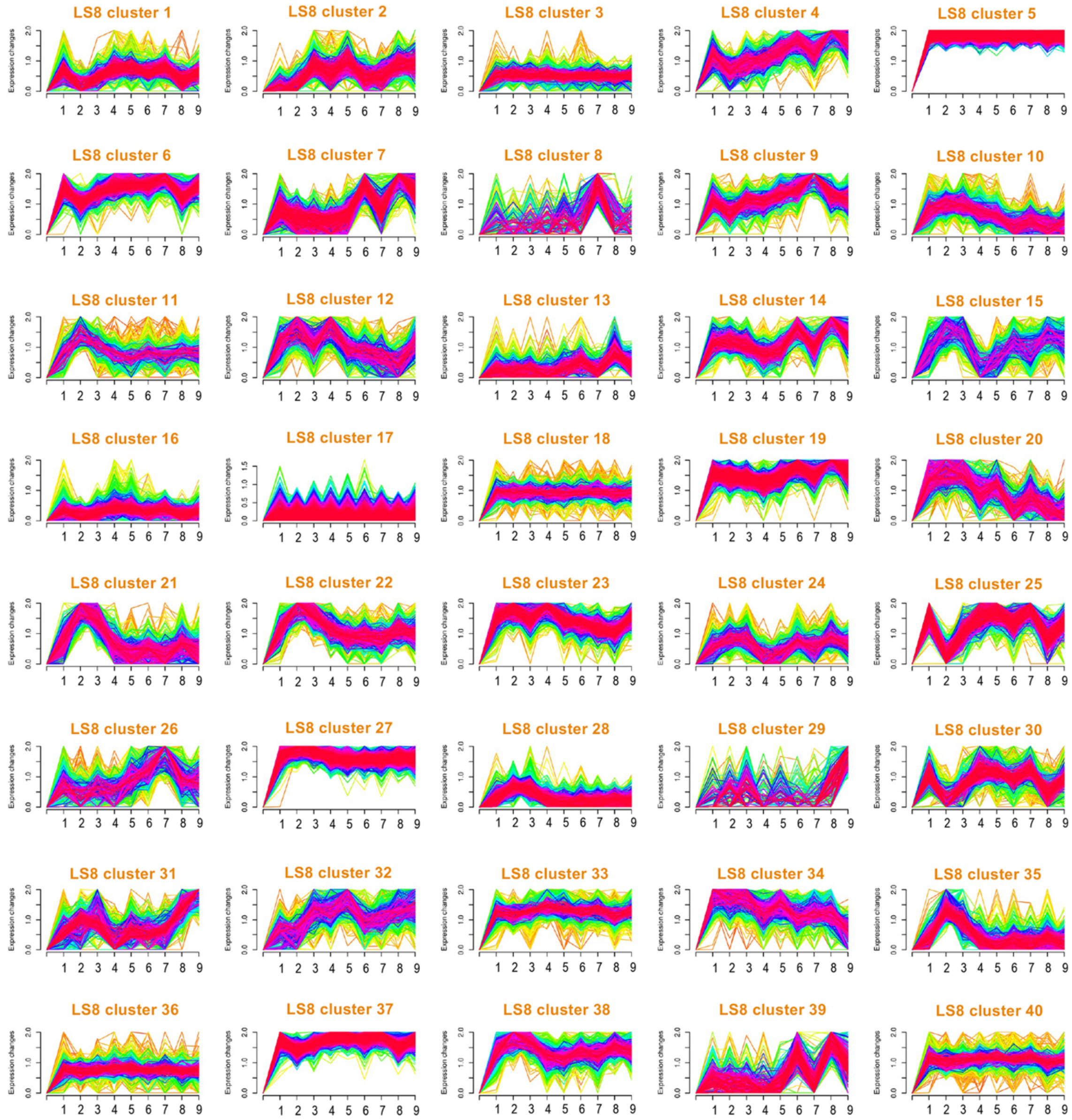

1	LS8_0
2	LS8_1C
3	LS8_3C
4	LS8_5C
5	LS8_13C
6	LS8_1T
7	LS8_3T
8	LS8_5T
9	LS8_13T

Supplementary Figure S2. Mfuzz analysis identified 40 different gene clusters in the LS8 RNA-seq samples. The representative expression trends are shown in each line chart. X axis represents the samples with sample names given on the right.

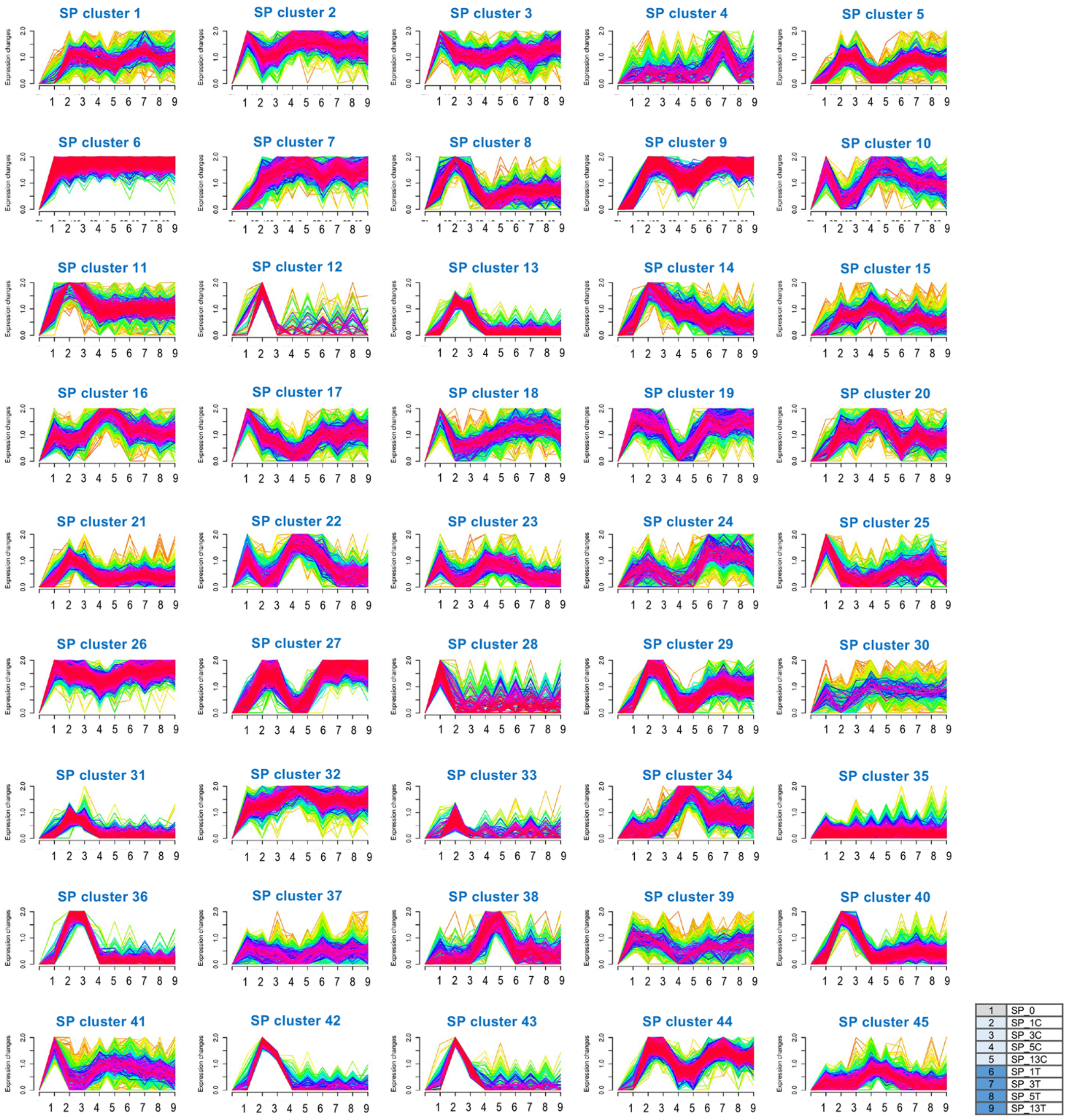

Supplementary Figure S3. Mfuzz analysis identified 45 different gene clusters in the SP RNA-seq samples. The representative expression trends are shown in each line chart. X axis represents the samples with sample names given on the right.

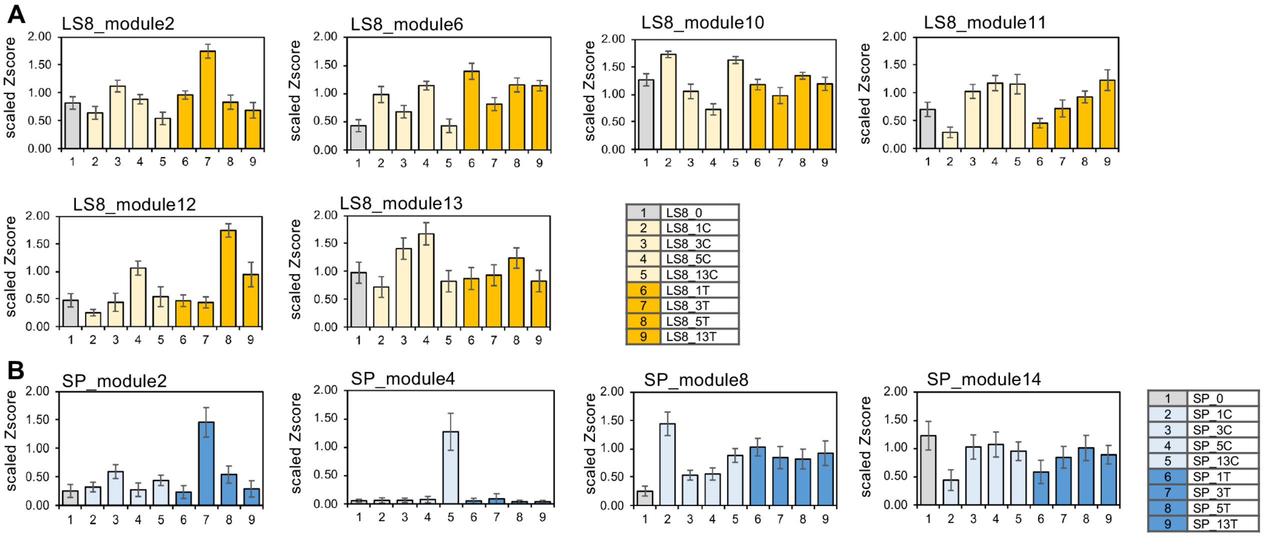

Supplementary Figure S4. The expression profiles of VW-inoculation associated modules in LS8 (A) and SP (B). X axis represents the samples with sample names given on the right.

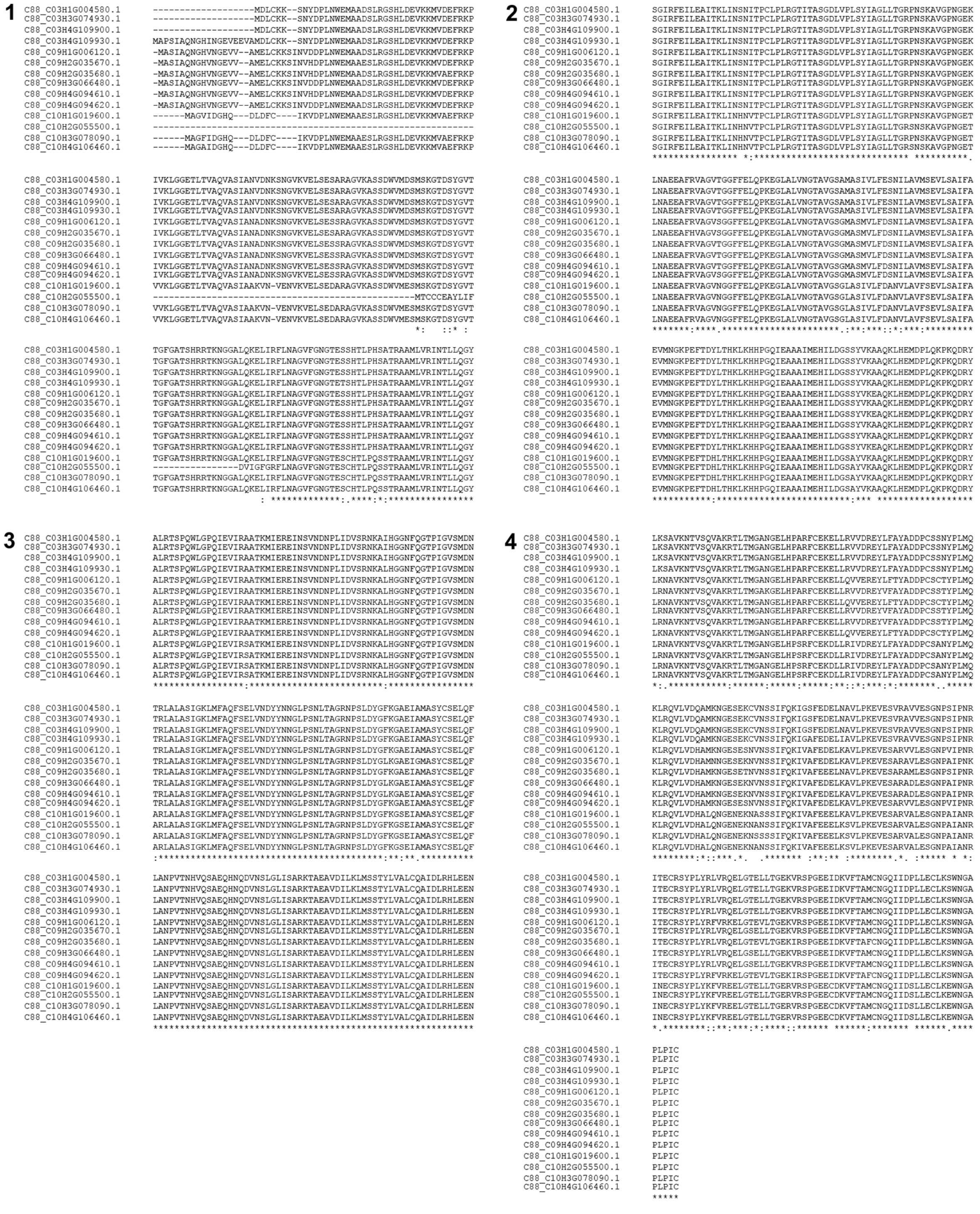

Supplementary Figure S5. StPAL protein sequence alignment with the MUSCLE algorithm. Aligned protein sequences were separated into four panels for visualization with each panel indicated with the corresponding numbers.
